# Hesperidin, a plant flavonoid accelerated the cutaneous wound healing in streptozotocin-induced diabetic rats: Role of TGF-ß/Smads and Ang-1/Tie-2 signaling pathways

**DOI:** 10.17179/excli2018-1036

**Published:** 2018-05-04

**Authors:** Wenbin Li, Amit D. Kandhare, Anwesha A. Mukherjee, Subhash L. Bodhankar

**Affiliations:** 1 Department of Dermatology, Shaanxi Traditional Chinese Medicine Hospital, Xi'an, Shaanxi, 710003, China; 2 Department of Pharmacology, Poona College of Pharmacy, Bharati Vidyapeeth Deemed University, Erandwane, Paud Road, Pune-411 038, India; 3 Jalan Universiti Bandar Barat, 31900, Kampar, Perak, Malaysia

**Keywords:** diabetic foot ulcer, hesperidin, VEGF-c, Ang-1, Tie-2, TGF-ß, Smad 2/3

## Abstract

**Background: **Delayed wound healing is a diverse, multifactorial, complex and inter-related complication of diabetes resulting in significant clinical morbidity. Hesperidin possesses potent antidiabetic and wound healing activity.

**Aim:** To evaluate the potential of hesperidin against experimentally induced diabetes foot ulcers.

**Methods:** Diabetes was induced experimentally by streptozotocin (STZ, 55 mg/kg, i.p.) in Sprague Dawley rats (180-220 g) and wounds were created on the dorsal surface of the hind paw of rats. Hesperidin (25, 50 and 100 mg/kg, p.o.) was administered for 21 days after wound stabilization. Various biochemical, molecular and histopathological parameters were evaluated in wound tissue.

**Results:** STZ-induced decrease in body weight and increase in blood glucose, food, and water intake was significantly (*p < *0.05) inhibited by hesperidin (50 and 100 mg/kg) treatment. It showed a significant increase (*p < *0.05) in percent wound closure and serum insulin level. The STZ-induced decrease in SOD and GSH level, as well as elevated MDA and NO levels, were significantly (*p < *0.05) attenuated by hesperidin (50 and 100 mg/kg) treatment. Intraperitoneal administration of STZ caused significant down-regulation in VEGF-c, Ang-1, Tie-2, TGF-β and Smad 2/3 mRNA expression in wound tissues whereas hesperidin (50 and 100 mg/kg) treatment showed significant up-regulation in these mRNA expressions. STZ-induced alteration in would architecture was also attenuated by hesperidin (50 and 100 mg/kg) treatment.

**Conclusion:** Together, treatment with hesperidin accelerate angiogenesis and vasculogenesis via up-regulation of VEGF-c, Ang-1/Tie-2, TGF-β and Smad-2/3 mRNA expression to enhance wound healing in chronic diabetic foot ulcers.

## Abbreviations

Angiopoietin-1 (Ang-1), Diabetes mellitus (DM), Diabetic wound control (DWC), Hematoxylin and Eosin (H&E), Lipid peroxidation (MDA), Nitric oxide (NO), Normal non-diabetic (ND), Normal wound control (NWC), Reactive oxygen species (ROS), Reduced glutathione (GSH), Reverse Transcriptase Polymerase Chain Reaction (RT-PCR), Standard Error of Mean (SEM), Streptozotocin (STZ), Superoxide dismutase (SOD), Transforming growth factor-β (TGF-β), Tumor necrosis factor-α (TNF-α), Vascular endothelial growth factor (VEGF-c)

## Introduction

Diabetes mellitus is a complex metabolic disorder affecting 171 million people worldwide, and this number is projected to reach 366 million by 2030 (Adil et al., 2017[1][6]; World Health Organization, 2016[120]). Improper control of diabetes mellitus leads to an array of macro- and microvascular complications such as cardiomyopathy, encephalopathy, neuropathy, nephropathy, retinopathy and foot ulcer (Badole et al., 2015[16]; Ghule et al., 2015[38]; Nathan, 1993[82]; Visnagri et al., 2014[116]). Diabetic neuropathy is the main risk factor associated with a diabetic foot ulcer, and this ulcer is a serious problem in clinical practice (Kandhare et al., 2012[68]; Rafehi et al., 2011[98]). It has been reported that amongst the diabetic patients 15 % will develop foot ulceration and wounds moreover 3 % of them will need amputation of a lower limb (Al-Watban et al., 2007[13]).

Normal wound healing is a well-orchestrated process characterized by four distinct inter-related phases including hemostasis, inflammation, proliferation, and remodeling (Diegelmann and Evans, 2004[32]; Patil et al., 2011[90]). Coordination between various cells, local release of growth factors and cytokines influence the rate of wound repair whereas disruption in this cycle leads to delayed wound healing (Goswami et al., 2016[43]; Shukla et al., 1997[106]). Diabetes mellitus is well known, and major contributors to delayed or impaired wound healing resulted in chronic ulcer formation. Several lines of studies suggested peripheral neuropathy and microvascular diseases are the vital factors that are delayed wound healing in diabetic condition (Ghosh et al., 2012[37]; Mustoe, 2004[80]; Shivakumar et al., 2014[105]).

Delayed wound healing exists with unknown etiology, and its underlying mechanisms are not yet completely understood. However, elevated oxidative stress, delayed collagen synthesis, decreased angiogenesis, impaired epithelialization and glucose metabolism, fibroblast and endothelial cell dysfunction has been reported as a vital pathophysiological factor in delaying the wound-healing process (Adil et al., 2017[7][6]; Bajpai et al., 2009[17]; Vijay et al., 2009[114]). Furthermore, increased apoptosis signaling and abnormal expression of some gap junction proteins, i.e., connexins also delayed the wound-healing process (Clark, 2008[26]). Numerous scientific reviews and studies demonstrate that inhibited production of transforming growth factor-β, insulin-like growth factor-1 and vascular endothelial growth factor resulted in delayed wound healing in diabetes mellitus (Perez Gutierrez and Vargas, 2006[95]; Teoh et al., 2009[113]). 

In spite of tremendous advances in the pharmaceutical drug industry, the treatments for diabetic wound ulcer have not progressed substantially in recent years. Nowadays, the approved the therapeutic implication for the treatment of diabetic foot ulcers are growth factor and cell therapies. However, their high cost and unwanted side effects limit their utility in the management of chronic wounds (Barrientos et al., 2014[18]; Kandhare et al., 2017[59]). Furthermore, as diabetic foot ulcer is a complex and multi-event clinical problem hence, it cannot be treated by single means, and there is a need of co-adjuvant therapies such as antidiabetic and antibiotics for glycemic and infection control. Moreover, it also needed daily wound care such as antiseptic bath as well as debridement and toe removal for gangrene depending upon need. Hence, there is an urge to the pursuit of new therapeutic agents for the treatment of non-healing diabetic foot ulcers. 

An array of compounds derived from natural plants (such as triterpenes, alkaloids, flavonoids) receives increasing acceptance (Kandhare et al., 2016[61]; Paocharoen, 2010[88]) due to non-toxic nature as well as they have an ability to influence one or more wound healing phases (Panchatcharam et al., 2006[87]; Sumitra et al., 2005[111]). In the light of the experimental evidence, it is documented that novel herbal moieties contain numerous phytoconstituents that can be explored to the management of chronic wounds. A large number of the popularly used ayurvedic plants have not been scientifically examined for their efficacy and anti-diabetic properties (Patel et al., 2012[89]). Furthermore, literature has indicated that animal models of impaired healing may have greater clinical relevance (Kandhare et al., 2014[57]; Nunan et al., 2014[84]) and played a vital role in the scientific evaluation of these unexplored moieties from the herbal origin. Since delayed diabetic wound healing is composite of multiple factors including impaired glycemic control, peripheral neuropathy, and lowered immunity against infections hence, Streptozotocin (STZ)-induced diabetic wound is an ideal model that represents these clinicopathological features of diabetic foot ulcer (Kandhare et al., 2011[53]).

Hesperidin (hesperetin-7-rhamnoglucoside) is a major plant flavonoid abundantly present in a variety of citrus species including *Citrus aurantium L*., *C. sinensis, C. unshiu* (Rutaceae) (Emim et al., 1994[33]; Kawaguchi et al., 1997[70]). Studies have shown hesperidin have many biological functions, including anti-diabetic, antihyperlipidemic, anti-ulcers, anti-inflammatory, anti-microbial, analgesic, antifungal, hepatoprotective, antioxidant, antiallergic, anti-cancer, anti-hypertensive and anti-atherogenic potential (Akiyama et al., 2010[11]; Jin et al., 2008[51]; Kandhare et al., 2017[60]; Kaur et al., 2006[69]; Kawaguchi et al., 1997[70]; Lee et al., 2004[74]; Nandakumar et al., 2011[81]; Shah and Patel, 2010[103]; Shi et al., 2012[104]). There is a report indicating that hesperidin ameliorates sodium arsenite-induced toxicity in rats (Pires Das Neves et al., 2004[97]).

Recently, the wound healing potential of hesperidin has been proven in experimental animal models and clinical trials (Ahmad et al., 2013[10]; Godeberge, 1994[39]; Hasanoglu et al., 2001[45]; Struckmann and Nicolaides, 1994[108]). The role of hesperidin in diabetes-induced complication such as diabetic nephropathy, neuropathy, cardiomyopathy, and encephalopathy have been proven effectively (Ibrahim, 2008[49]; Shah and Patel, 2010[103]). To best of our knowledge, no study has been carried out that evaluated the effect of hesperidin in the diabetic wound. Hence, the aim of present investigation to evaluate the potential of administration of hesperidin against experimentally induced diabetes foot ulcers.

## Material and Method

### Animals

Adult Sprague Dawley rats (male, 180-220 g) were obtained from the National Institute of Biosciences, Pune (India). Rats were housed at 24 ± 1 °C, with a relative humidity of 45-55 % and 12:12 h dark/light cycle. The animals had free access to standard pellet chow (Pranav Agro Industries Ltd., Sangli, India) and filtered water throughout the experimental protocol. All experiments were carried out between 09:00 and 17:00 h. The experiment was conducted according to the guidelines of Committee for Control and Supervision of Experimentation on Animals (CPCSEA), Government of India on animal experimentation. 

### Chemicals

Hesperidin and Streptozotocin (Sigma Chemical Co., St Louis, MO, USA), Total RNA Extraction kit (MP Biomedicals India Private Limited, India) and One-step Reverse transcription-polymerase chain reaction (RT-PCR) kit (MP Biomedicals India Private Limited, India), GOD‐POD (glucose oxidase-peroxidase) diagnostic kit (Accurex Biomedical Pvt. Ltd., Mumbai, India) were procured from respective manufacturer.

### Induction and assessment of diabetes

Diabetes was induced by intraperitoneal administration of streptozotocin (STZ, 55 mg/kg) in citrate buffer (pH 4.4, 0.1 M) (Visnagri et al., 2012[118]). A separate group of age‐matched control rats were also maintained which received an equal volume of citrate buffer. Retro-orbital plexus technique was used to collect the blood samples, and GOD‐POD was used to estimate serum glucose levels. Rats having serum glucose levels more than 250 mg/dL were selected as diabetic and used for the present study. 

### Excision wound model

Excision diabetic foot ulcer was created according to the previously established method (Kandhare et al., 2014[57]). Briefly, on the day of wound creation, each rat was anesthetized with ketamine (75 mg/kg, i.p.) and xylazine (10 mg/kg, i.p.). The rectangular wound (2 mm × 5 mm) was created on the dorsal surface of the foot of each rat. The rats were randomly divided into various experimental groups.

### Experimental design

After wound creation, rats were divided into following experimental groups (n=6, each):

#### A. Non-diabetic and non-wounded animals

**Group I:** Normal non-diabetic (ND): non-diabetic animals without wound received double distilled water (10 mg/kg, p.o.) for 21 days.

#### B. Non-diabetic and wounded animals

**Group II:** Normal wound control (NWC): non-diabetic animals with wound received a single injection of citrate buffer (vehicle), and then double distilled water (10 mg/kg, p.o.) for 21 days.

#### C. Diabetic and wounded animals

**Group III:** Diabetic wound control (DWC): diabetic animals with wound received double distilled water (10 mg/kg, p.o.) for 21 days.

**Group IV:** Hesperidin (H (25)): diabetic animals with wound received hesperidin (25 mg/kg, p.o.) for 21 days.

**Group V:** Hesperidin (H (50)): diabetic animals with wound received hesperidin (50 mg/kg, p.o.) for 21 days.

**Group VI:** Hesperidin (H (100)): diabetic animals with wound received hesperidin (100 mg/kg, p.o.) for 21 days.

**Group VII:** Insulin (I (10)): diabetic animals with wound received insulin (10 IU/kg, s.c.) for 21 days.

Every day the three different dosages of hesperidin, i.e., 25, 50 and 100 mg/kg were freshly and administered to animals for 21 days. On various days, the wound area was determined with the help of digital camera (Fuji, S20 Pro, Japan) by an observer blind to the treatment. The metabolic cages (Techniplast, Italy) were used to determine food intake and water intake. 

After 22 days, rats were anesthetized under etheral anasthesia and blood was withdrawn by a retro-orbital puncture for determination of serum parameters. Then, animals were sacrificed by cervical dislocation and wound tissues were rapidly removed and stored at -80 °C for biochemical parameters.

### Measurement of the wound area, calculation of wound contraction and determination of half-closure time (CT50)

On day various days including 4, 8, 12, 16 and 21, the progressive changes in wound area were recorded using camera (Fuji, S20 Pro, Japan). Then, the wound area was determined using image analysis software (Image J, NIH, USA). 

The percent (%) wound closure was calculated by following formula: % Wound closure = 100 * [(Initial wound area) - (N^th^ day wound area)/ (Initial wound area)]. 

The graph of percent wound closure versus time in days from wound creation was plotted using the software GraphPad Prism v5.0, and linear regression analysis was performed. The value of X corresponds to Y=50 % was consider as CT50 (half-closure time, the time taken to close the wound by 50 %).

### Determination of serum insulin

The serum was separated by centrifugation (4 °C, 5200 *g*, 15 min) using Eppendorf Cryocentrifuge (model No. 5810, Germany). The assay of serum insulin was performed by a rat ELISA (enzyme-linked immunosorbent assay) kit (Mercodia AB, Uppsala, Sweden).

### Biochemical estimations

#### Preparation of tissue homogenate

Skin tissue segments were weight and mixed with 0.1 M phosphate buffer (pH 7.4) and homogenized on ice for 60 seconds at 10600 *g* (Remi Equipment Pvt. Ltd., Remi Motors Ltd., Mumbai, India). Supernatant of tissue homogenates was used to determine superoxide dismutase (SOD), reduced glutathione (GSH), lipid peroxidation (MDA), nitric oxide (NO), and hydroxyproline content as described previously (Adil et al., 2016[4]; Honmore et al., 2015[47]; Kandhare et al., 2012[66][67], 2013[58], 2015[55]; Mohod et al., 2016[76]; Mukherjee et al., 2015[78]).

### Reverse transcriptase PCR analysis of VEGF-c, Ang-1, Tie-2, TGF-β and Smad 2/3 mRNA expression

The reverse transcription (RT)-PCR used to determine mRNA levels in skin tissue (Adil et al., 2016[2]; Kandhare et al., 2013[58], 2015[64]; Mohod et al., 2016[76]). Briefly, total RNA was extracted from skin tissues according to the manufacturer's instructions (MP Biomedicals India Private Limited, India). The polymerase chain reaction mixture was amplified in a DNA thermal cycler (Eppendorf India Ltd, Chennai, India) by using gene-specific primers (Table 1(Tab. 1)). PCR products were run on 1 % agarose gel, stained with ethidium bromide. Gene expression was assessed by generating densitometry data for band intensities in different sets of experiments, by analyzing the gel images on the Image J program (Version 1.33, Wayne Rasband, National Institutes of Health, Bethesda, MD, USA). The band intensities were compared with constitutively expressed β-actin which served as a control for sample loading and integrity. The intensity of mRNAs was standardized against that of the β-actin mRNA from each sample, and the results were expressed as PCR-product/β-actin mRNA ratio.

### Histopathological examination

Another portion of skin tissue was stored in 10 % formalin for 24 h. The specimen was dehydrated and placed in xylene for 1 h (3 times) and later in ethyl alcohol (70, 90 and 100 %) for 2 h respectively. The infiltration and impregnation were carried out by treating with paraffin wax twice, each time for 1 h. Tissue specimens were cut into sections of 3-5 µm thickness and were stained with hematoxylin and eosin (H&E). The specimen was mounted on the slide by use of DPX (distrene, with dibutyl phthalate and xylene) as mounting medium. Sections were examined under a light microscope to obtain a general impression of the histopathology features of specimen and infiltration of cells. The various changes in histological features were graded as Grade 0 (not present); Grade 1 (mild); Grade 2 (moderate); and Grade 3 (severe) as described previously (Patil et al., 2012[93]).

### Statistical analysis

Data are expressed as mean ± standard error mean (SEM). Data analysis was performed using Graph Pad Prism 5.0 software (Graph Pad, San Diego, CA, USA). Data of wound area and percentage wound closure were analyzed using two-way repeated analysis of variance (ANOVA), and Bonferroni's multiple range test was applied for post hoc analysis, while data of biochemical parameters were analyzed using one-way analysis of variance (ANOVA), and Tukey's multiple range test was applied for post hoc analysis. A value of *p < *0.05 was considered to be statistically significant.

## Results

### Effect of hesperidin and insulin treatment on body weight, serum glucose level and serum insulin of diabetic rats

There was a significant decrease (*p < *0.05) in body weight and serum insulin level whereas an increase in serum glucose level of vehicle control rats after induction of diabetes by intraperitoneal administration of STZ. Treatment with hesperidin (50 and 100 mg/ kg) significantly (*p < *0.05) inhibit STZ-induced decreased in body weight and serum insulin level as compared to diabetic wound control. An elevated level of serum glucose level also significantly (*p < *0.05) decreased by hesperidin (50 and 100 mg/kg) as compared to diabetic wound control (Table 2(Tab. 2)). Administration of insulin (10 IU/kg) also significant (*p < *0.05) increased body weight and serum insulin level whereas significantly decreased (*p < *0.05) serum glucose level as compared to diabetic wound control. Insulin (10 IU/kg) also showed more significant decrease (*p < *0.05) in serum glucose level and significant increase (*p < *0.05) in serum insulin level as compared to hesperidin (25, 50 and 100 mg/kg) treatment (Table 2(Tab. 2)).

### Effect of hesperidin and insulin treatment on food and water intake of diabetic rats

The food and water intake were increased significantly (*p < *0.05) in diabetic wound control rats after intraperitoneal administration of STZ. Treatment with hesperidin (50 and 100 mg/kg) significantly (*p < *0.05) reduced the food and water intake when compared with diabetic wound control rats. Diabetes-induced increase in food and water intake was also significantly (*p < *0.05) reduced by insulin treatment (10 IU/kg) when compared with diabetic wound control rats. Insulin (10 IU/kg) treatment showed more significant (*p < *0.05) improvement to the altered food and water intake as compared to hesperidin (25 and 50 mg/kg) treated rats (Table 2(Tab. 2)).

### Effect of hesperidin and insulin treatment on rate of wound contraction and CT50 of diabetic rats

**Figure 1:**
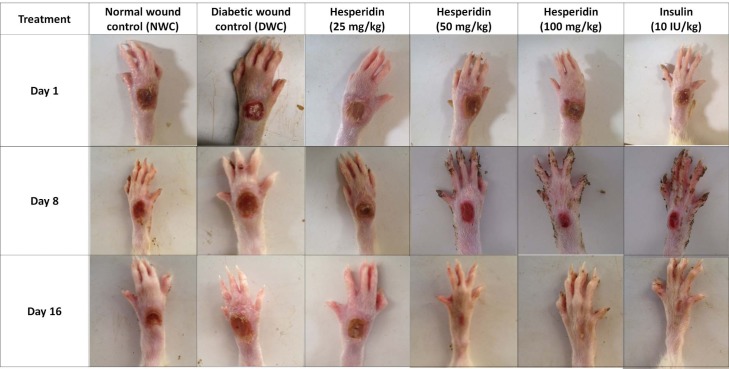
Morphological representation of rat paws showing various phases of wound healing

The effect of hesperidin and insulin treatment on the morphology of various stages of wound healing is shown in Figure 1(Fig. 1). Diabetic wound control rats showed a higher wound area where the rate of wound contraction was lower over a period of 21 days. The wound area was significantly decreased (*p < *0.05), and the rate of wound contraction was significantly (*p < *0.05) increased by hesperidin (50 and 100 mg/kg) treatment when compared with diabetic wound control rats. The wound area and rate of wound contraction were also significantly ameliorated (*p < *0.05) by treatment with insulin (10 IU/kg) as compared to diabetic wound control rats (Table 2(Tab. 2)). Furthermore, the rate of wound contraction was significantly higher (*p < *0.05) in hesperidin (100 mg/kg) treated rats as compared to insulin (10 IU/kg) treated rats (Table 2(Tab. 2)). 

The CT50 value for diabetic wound control rats was -272.6 days (negative sign indicated delayed wound healing). CT50 value for the combination of hesperidin (25, 50 and 100 mg/kg) treated rats was 67.95, 22.78 and 13.78 days respectively whereas, CT50 values for insulin (10 IU/kg) treated rats was 21.77 days (Table 2(Tab. 2)).

### Effect of hesperidin and insulin treatment on the oxido-nitrosative stress of diabetic rats

The SOD and GSH levels decreased significantly (*p < *0.05) and MDA and NO levels increased significantly (*p < *0.05) in normal wound control rats and diabetic wound control rats. Treatment with hesperidin (50 and 100 mg/kg) significantly (*p < *0.05) reduced diabetes-induced alterations in oxido-nitrosative stress when compared with diabetic wound control rats. Insulin treatment (10 IU/kg) also significantly (*p < *0.05) inhibited diabetes-induced alteration in SOD, GSH, MDA and NO levels as compared to diabetic wound control rats (Table 3(Tab. 3)).

### Effect of hesperidin and insulin treatment on hydroxyproline level of diabetic rats

Diabetic wound control rats showed significant (*p < *0.05) decrease in hydroxyproline level as compared to normal wound control as well as normal control rats. When compared with diabetic wound control rats, hesperidin (50 and 100 mg/kg) treatment significantly (*p < *0.05) increased hydroxyproline level. Hydroxyproline level also significantly (*p < *0.05) increased by treatment with insulin (10 IU/kg) as compared with diabetic wound control rats. When compared with insulin (10 IU/kg) treatment, hesperidin (50 and 100 mg/kg) treated rats showed significantly higher (*p < *0.05) hydroxyproline level (Table 3(Tab. 3)). 

### Effect of hesperidin and insulin treatment on VEGF-c, Ang-1, Tie-2, TGF-β and Smad 2/3 mRNA expression in wound skin tissue of diabetic rats

**Figure 2:**
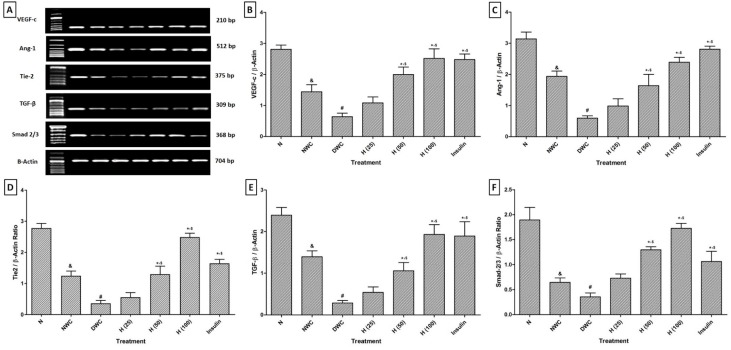
Effect of hesperidin and insulin treatment on mRNA expression of VEGF-c, Ang-1, Tie-2, TGF-β and Smad 2/3in wound tissue as determined by relative quantification by reverse transcriptase polymerase chain reaction analysis (A), Quantitative representation of mRNA expression of VEGF-c, Ang-1, Tie-2, TGF-β and Smad 2/3 (B-F). Data are expressed as mean ± SEM (n = 6) and analyzed by one-way ANOVA followed by Tukey's multiple range test for each parameter separately. ^&^*p < *0.05 as compared to normal, ^#^*p < *0.05 as compared to normal wound control, *p < 0.05 as compared to diabetic wound control group and ^$^*p < *0.05 as compared to one another. NWC: Normal wound control group; DWC: Diabetic wound control group; I (10): Insulin (10 IU/kg, s.c.) treated group; H (25): Hesperidin (25 mg/kg, p.o.) treated group; H (50): Hesperidin (50 mg/kg, p.o.) treated group; H (100): Hesperidin (100 mg/kg, p.o.) treated group.

There was down-regulation in VEGF-c, Ang-1, Tie-2, TGF-β and Smad 2/3 mRNA expression in skin tissue of normal wound control rats as well as diabetic wound control rats. The mRNA expression of VEGF-c, Ang-1, Tie-2, TGF-β and Smad 2/3 were up-regulated significantly (*p < *0.05) in hesperidin (50 and 100 mg/kg) treatment when compared to diabetic wound control rats. Insulin (10 IU/kg) treatment also significantly inhibited (*p < *0.05) STZ-induced down-regulation in VEGF-c, Ang-1, Tie-2, TGF-β and Smad 2/3 mRNA expression as compared to diabetic wound control rats. There was a significant (*p < *0.05) up-regulation in VEGF-c, Tie-2, TGF-β and Smad 2/3 mRNA expression in the hesperidin (100 mg/kg) treated group as compared to insulin (10 IU/kg) treated rats (Figure 2(Fig. 2)).

### Effect of hesperidin and insulin treatment on diabetes-induced histopathological alterations in skin tissue of diabetic rats

**Figure 3:**
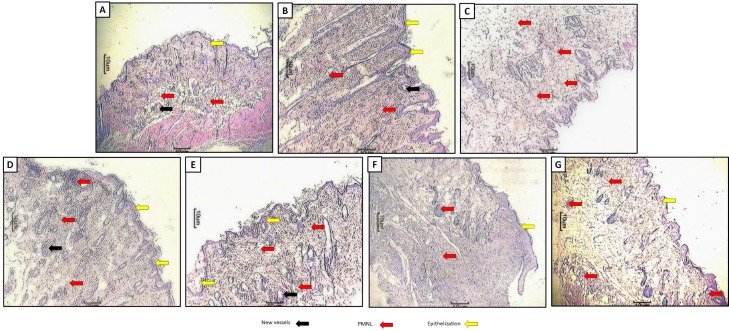
Photomicrographs of sections of Hematoxylin & eosin-stained wound skin tissues. Microscopic image of wound skin of normal control rats (A), normal wound control rat (B), diabetic (STZ) wound control group (C), hesperidin (25 mg/kg, p.o.)-treated group (D), hesperidin (50 mg/kg, p.o.)-treated group (E), hesperidin (50 mg/kg, p.o.)-treated group (F) and insulin (10 IU/kg, s.c.)-treated group (G). Formation of new vessels (black arrow), the formation of epithelization (yellow arrow) and inflammatory infiltration (red arrow). Images (×100 magnification) are typical and representative of each study group.

Figure 3A(Fig. 3) represented the normal architecture of normal tissue from normal control rats. It showed the presence of vessels, epithelial layer with few polymorphonuclear leukocyte infiltrations (red arrow, grade 1). Figure 3B(Fig. 3) showed the distorted architecture of skin from normal wound control rats. Wound tissue from diabetic control rats showed the presence of ectopic vessels with edema along with polymorphonuclear leukocyte infiltration (red arrow, grade 4). It also showed thicker epithelial layer and disorganized fibroblasts (Figure 3C(Fig. 3)). Skin tissue from insulin (10 IU/kg) treated rats showed well-organized dermal layers with decreased polymorphonuclear leukocyte infiltration (red arrow, grade 2) with re-epithelialization (yellow arrow, grade 3) and new vessels formation (black arrow, grade 3) (Figure 3D(Fig. 3)). Wound tissue from the hesperidin (25 mg/kg) treated rats demonstrated incomplete epithelialization (grade 1), lesser number new vessels (grade 1), and polymorphonuclear leukocyte infiltration (grade 3) (Figure 3E(Fig. 3)). Histopathology of wound skin from hesperidin (50 and 100 mg/kg) treated rats revealed an increased number of blood vessels (grade 3), re-epithelialization (grade 3) with mild polymorphonuclear leukocyte infiltration (grade 1) (Figure 3F(Fig. 3) and Figure 3G(Fig. 3), respectively) (Table 4(Tab. 4)).

See also the Supplementary Data.

## Discussion

Delayed wound healing is a diverse, multifactorial, complex and inter-related complication of diabetes resulting in significant clinical morbidity. Common causes of diabetic foot ulcer include elevated blood sugar that leads to impaired blood flow and oxygen release, protein malnutrition that causes decreased collagen and fibronectin synthesis, decreased local immune and cell defenses and impaired insulin and growth hormone (Adil et al., 2017[8]; Reiser, 1991[101]). Moreover, it has been documented that hyperglycemia leads to dysfunctioning of neutrophil which includes migration, chemotaxis, adherence and phagocytic and bactericidal potential (Wall et al., 2003[119]). Wound healing process includes a sequence of biological events including wound closure, repair, and remodeling of damaged tissue (Phillips et al., 1991[96]). However, the presence of hyperglycemia causes delayed wound healing, and therefore it led to the development of many pharmacological treatments. The current treatment regimen is able to show the effect on only one of the underlying cause, and they are also associated with undesirable side effects (Suh et al., 1998[110]). Hence, there is a need for safer and more effective healing agents which shows effect via acting through more than one mechanism in diabetic foot ulcer. In the view of this, we have investigated the effect of administration of hesperidin in STZ-induced diabetic foot ulcer by evaluating various biochemical and molecular parameters.

Researcher revealed that decrease body weight provided insight to disease state (Kamble et al., 2013[52]; Tambewagh et al., 2017[112]). In the present study, intraperitoneal administration of STZ caused a significant decrease in body weight, and this observation is similar to earlier reports (Kandhare et al., 2014[57], 2017[59]). In the past, researchers have underlined the correlation between diabetes and decrease in the availability of glucose and amino acid. The impaired cellular biosynthesis and metabolism are considered as an underlying cause of unavailability of glucose and amino acid which is induced by diabetes (Adil et al., 2014[9], 2017[1]). In agreement with other literature, the finding of the present study showed that intraperitoneal administration of STZ resulted in features like polydipsia, and polyphagia (Kandhare et al., 2014[57]) whereas administration of hesperidin significantly attenuated this diabetes-induced polydipsia, and polyphagia.

Studies have shown a predominant role for fibroblasts during wound healing process (Etscheid et al., 2004[34]). Contraction of the wound is an essential phenomenon in healing process which mediated via fibroblasts within the granular tissue by differentiation of fibroblasts into myofibroblasts (Sidhu et al., 1999[107]). This fibroblast proliferation leads to increased collagen production (Chen et al., 2005[23]). The presence of myofibroblasts in the healing area is considered as an important feature of tissue undergoing contraction (Moulin et al., 2000[77]). Researchers previously reported that hesperidin treatment enhances wound healing in non-diabetic rats (Ahmad et al., 2013[10]; Godeberge, 1994[39]; Hasanoglu et al., 2001[45]; Struckmann and Nicolaides, 1994[108]). In the present investigation, administration of hesperidin showed enhanced wound healing which may be due to its potential for increased fibroblasts proliferation. 

Angiogenesis plays a crucial role in homeostasis and development of organs (Heinke et al., 2012[46]). Furthermore, in adults, the important integral feature of angiogenesis in wound healing, ischemic diseases recovery and tissue regeneration (Navaratna et al., 2009[83]). Its dysregulation leads to the pathogenesis of an array of maladies including microvascular complications (neuropathy and retinopathy), cancer, and arthritis (Bhilare et al., 2016[20]; Gosavi et al., 2011[41]; Kandhare et al., 2016[54]; Patil et al., 2012[91]). The process of angiogenesis leads to the generation of new vessels from pre-existing ones that help to alleviate some states including healing of wounds through delivering oxygen and nutrients (Crivellato, 2011[28]). Numerous scientific reviews and studies demonstrate that recent therapies caused stimulation of only one angiogenic factor (e.g., vascular endothelial growth factor (VEGF-c)) (Navaratna et al., 2009[83]). However, nowadays it is becoming a need that therapeutic moiety should be enhanced angiogenesis through modulation of various angiogenic factors and their receptors (Carmeliet and Jain, 2011[22]; Chung and Ferrara, 2011[25]). A compelling body of evidence supports that angiogenesis delayed by hyperglycemia (Bek et al., 2002[19]; da Costa Pinto and Malucelli, 2002[29]). Therefore, it is important to reduce hyperglycemia along with an increase in angiogenesis for the treatment of diabetic foot ulcer healing (Frykberg and Banks, 2015[36]). Interestingly, in the present study, treatment with hesperidin significantly decreased blood sugar level and also increased angiogenesis. This possible interplay between wound contraction and hyperglycemia by hesperidin treatment may accelerate the diabetic wound healing process.

Although the precise mechanism of foot ulcer caused by diabetes is not clear, there is evidence that elevated blood sugar level can cause generation of reactive oxygen metabolites and inhibit the activity of antioxidant enzymes in tissue (Kandhare et al., 2012[66], 2017[59]; Visnagri et al., 2014[116]). Numerous literature have supported the direct link between oxidative stress and diabetic foot ulcer (Bajpai et al., 2009[17]; Kandhare et al., 2014[57]). Oxidative stress is generated via reactive oxygen species (ROS) that plays an important role in down-regulation of endogenous antioxidant enzymes such as SOD and GSH (Adil et al., 2014[9]; Aswar et al., 2015[15]; Kandhare et al., 2015[56][62]; Ketkar et al., 2015[71]). Furthermore, these elevated levels of ROS also associated with elevated levels of lipid peroxidation (i.e., malonaldehyde or MDA) which destroyed membrane lipid bilayer arrangement (Mukherjee et al., 2017[79]; Patil et al., 2012[92]; Raygude et al., 2012[100]; Visnagri et al., 2013[117]). These consequences caused an alteration in tissue permeability and deactivation of membrane-bound receptors. Additionally, excessive synthesis of NO plays a key role in oxidative damage via interacting with superoxide to generate the peroxynitrite (Kandhare et al., 2016[65]; Kumar et al., 2014[73]; Patil et al., 2012[94]). It has been suggested that antioxidants may play an important role in abating some hazards which are associated with elevated oxido-nitrosative stress (Devkar et al., 2016[31]; Gosavi et al., 2012[41]; Kandhare et al., 2017[63]). In the present investigation, hesperidin exerts an antioxidant effect through scavenging free radicals or via activating/inducing cellular antioxidant enzyme systems. Numerous evidence supported the antioxidant potential of hesperidin overproduction of free radicals in an array of disease which is associated with depletion of antioxidant enzymes such as SOD, GSH, MDA and NO (Kandhare et al., 2017[60]; Visnagri et al., 2014[116]). Results of the present investigation are in accordance with the findings of the previous investigator.

Angiopoietin 1 (Ang-1), a type of 70 kDa glycoprotein from angiopoietin family, serves as a ligand of the Tie receptor tyrosine kinase (Pafumi et al., 2015[86]). The phosphorylation of the Tie-1 receptor by Ang-1 initiates Ang-1-Tie signaling, and this Ang-1-Tie interaction resulted in its expression in endothelial cells where it plays a vital role in angiogenesis for development and regeneration (Dallabrida et al., 2005[30]; Jeansson et al., 2011[50]). Cho et al. (2005[24]) reported the accelerated wound healing via angiogenesis by application of recombinant adenovirus encoding angiopoietin-1 (Ad-Ang-1). Thus, a moiety which has an ability to activate Ang-1-Tie signaling that serves as a promising therapeutic candidate for effective angiogenesis and vascular protection (Koh, 2013[72]). In the present study, treatment with hesperidin caused up-regulation in Ang-1-Tie resulted in increased angiogenesis leading to accelerated wound healing via stabilized blood vessel integrity and vascular remodeling. Results of the present investigation are in line with the findings of the previous investigation which showed the angiogenic potential of hesperidin (Haddadi et al., 2017[44]). 

Vascular endothelial growth factor (VEGF-c) is a cytokine that is produced by numerous cell types such as keratinocytes, macrophages, and fibroblasts (Altavilla et al., 2001[12]). VEGF-c is responsible for endothelial cell survival, proliferation, and migration. It has been previously described that vasculogenesis also plays an important role in the healing process along with angiogenesis (Visconti et al., 2002[115]). It is also reported that VEGF-c is an endogenous stimulator of angiogenesis (Corral et al., 1999[27]). Furthermore, angiogenesis is associated with expression of cytokines and angiogenic factors which includes VEGF-c (Ferrara et al., 2003[35]). So collectively, both angiogenesis and vasculogenesis can be accelerated by activation of VEGF-c and Ang-1 respectively. In diabetic condition, the VEGF-c level was decreased and resulted in increased activity of gap junction. This causes increased apoptotic, pro-inflammatory, and toxic signaling pathways from injured part to adjacent healthy parts through gap junctions which resulted in impaired wound healing process (Suarez and Ballmer-Hofer, 2001[109]). In the light of this report, our results also showed delayed wound healing in diabetic animals. Whereas, treatment with hesperidin showed improvement in the altered healing process via enhancement in the impaired angiogenesis through Ang-1/VEGF-c dependent manner. The previous investigator also showed a significant increase in VEGF-c level in the rat skin tissue *in-vivo* after treatment with hesperidin (Haddadi et al., 2017[44]). Results of the present investigation agreed with the findings of the previous researcher.

An array of evidence suggests that diabetes is associated with various connective tissue abnormalities. Collagen is an important extracellular connective tissue protein that provides strength and integrity to the tissue matrix (Raghow, 1994[99]). Abnormalities in the synthesis of this connective tissue protein at the wound area after an injury delays healing process (Nwomeh et al., 1999[85]). Hyperglycemia is one the factors that interfere with collagen synthesis. Thus, delays wound healing in diabetes (Goodson III and Hunt, 1977[40]). Hydroxyproline is served as a hallmark of collagen turnover (Ricard-Blum and Ruggiero, 2005[102]). It is well documented that collagen synthesis can be accelerated by the process of angiogenesis which increases the delivery of oxygen and other nutrients at the site of local injury to elevate the local collagen turnover (Buemi et al., 2002[21]). Furthermore, simultaneous stimulation of collagen and VEGF-c synthesis have also been reported beneficial during delayed wound healing (Kandhare et al., 2014[57]). In congruence with the literature, our findings show that treatment with hesperidin caused increased angiogenesis, VEGF-c and collagen synthesis that may result in accelerated wound healing in diabetic foot ulcer.

It has already been known that Smad2 and Smad3 dissociate from the receptor complex and form another heteromeric complex with Smad4. These complexes translocate into the nucleus and trigger pro-fibrogenic genes that are amenable to TGF-β (Adil et al., 2015[5], 2016[3]). In the current study, intraperitoneal administration of STZ caused noteworthy down-regulation of TGF-β expression in wound tissues as compared to normal wound rats which are concurrent with the previous reports (Hozzein et al., 2015[48]). For instances, the body of evidence showed that considerably increased TGF-β protein expression, and Smad-2/3 phosphorylation is positively linked to fibrosis through TGF-β/Smad pathway in diabetic foot ulcer (Xu et al., 2013[122]). In the past, it has been reported that amplified TGF-β and Smad-2/3 expression for wound tissue is in accordance with elevated deposition of collagens (Xu et al., 2013[122]). Taken our current data together with evidence from other groups, it can be concluded that diabetic induced foot ulcer is strongly allied with decreased TGF-β and Smad-2/3 mRNA expression. However, administration of hesperidin significantly upregulates TGF-β and Smad-2/3 mRNA expression in wound tissue. The result of the present investigation is in accordance with the findings of the previous researcher (Wu et al., 2015[121]). 

Recently, flavonoids have been reported efficacious and cost-effective in the management of chronic foot ulcers (Antunes-Ricardo et al., 2015[14]). Furthermore, two herbal formulae were shown its wound healing potential against chronic diabetic ulcers in a randomized, double-blind, placebo-controlled trial (Leung et al., 2008[75]). These remarkable a series of scientific evidence may open novel vistas for the therapeutic option of diabetic foot ulcers with natural remedies like hesperidin. In conclusion, results of present investigations showed that hesperidin treatment inhibited diabetes-induced increased blood glucose level and down-regulated VEGF-c, Ang-1/Tie-2, TGF-β and Smad-2/3 mRNA expression to accelerate angiogenesis and vasculogenesis resulted in enhances wound healing in chronic diabetic foot ulcers.

## Conflict of interest

The authors declare no conflict of interest. 

## Supplementary Material

Supplementary data

## Figures and Tables

**Table 1:**
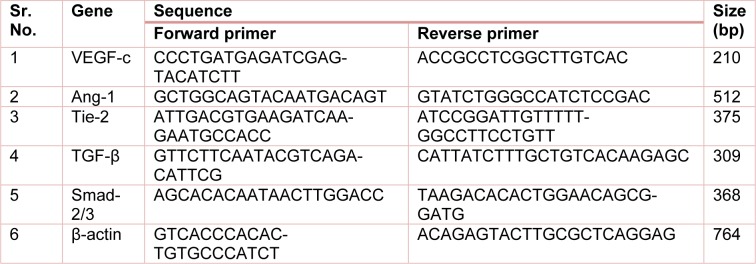
Primer sequences for VEGF-c, Ang-1, Tie-2, TGF-β, Smad 2/3, and β-actin

**Table 2:**
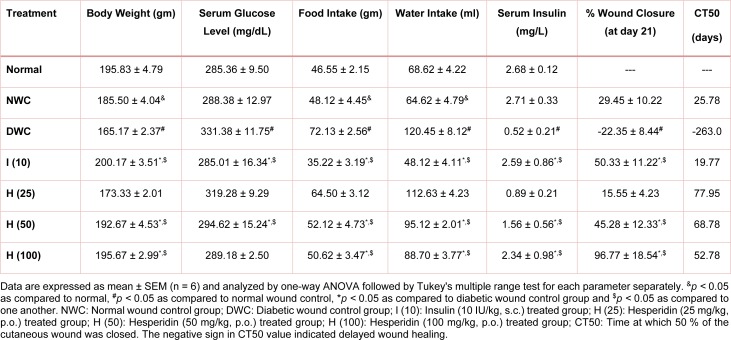
Effect of hesperidin and insulin treatment on body weight, serum glucose level, food intake, water intake, serum insulin and CT50 of diabetic rats

**Table 3:**
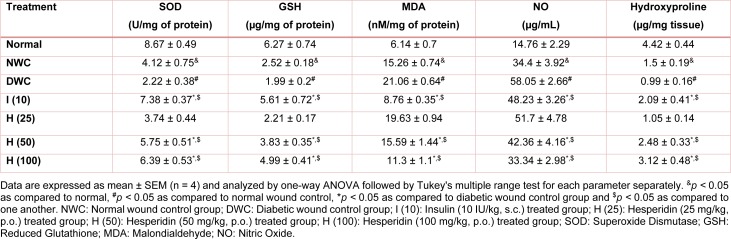
Effect of hesperidin and insulin treatment on the oxido-nitrosative stress and hydroxyproline level of diabetic rats

**Table 4:**
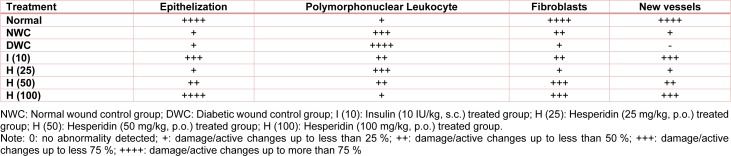
Effect of hesperidin and insulin treatment on wound healing processes and healing phases of diabetic rats
