# Supplementary material for: Hesperidin, a plant flavonoid accelerated the cutaneous wound healing in streptozotocin-induced diabetic rats: Role of TGF-ß/Smads and Ang-1/Tie-2 signaling pathways
Source: EXCLI J. 2018 May 4;17:399–419. doi: 10.17179/excli2018-1036 (PMC5962903; doi:10.17179/excli2018-1036)
Supplement: Supplementary data [file EXCLI-17-399-s-001.pdf]

EXCLI Journal 2018;17:399-419 – ISSN 1611-2156
Received: January 07, 2018, accepted: March 28, 2018, published: May 04, 2018

Supplementary data to:

HESPERIDIN, A PLANT FLAVONOID ACCELERATED THE CUTANEOUS WOUND HEALING IN STREPTOZOTOCIN-INDUCED DIABETIC RATS: ROLE OF TGF-Β/SMADS AND ANG-1/TIE2 SIGNALING PATHWAYS

Wenbin Li[1], Amit D. Kandhare[2,3]*, Anwesha A. Mukherjee[2], Subhash L. Bodhankar[2]

[1] Department of Dermatology, Shaanxi Traditional Chinese Medicine Hospital, Xi’an, Shaanxi, 710003, China
[2] Department of Pharmacology, Poona College of Pharmacy, Bharati Vidyapeeth Deemed University, Erandwane, Paud Road, Pune-411 038, India
[3] Jalan Universiti Bandar Barat, 31900, Kampar, Perak, Malaysia

* Corresponding author: Dr. Amit D. Kandhare, Department of Pharmacology, Poona College of Pharmacy, Bharati Vidyapeeth Deemed University, Erandwane, Paud Road, Pune-411 038, India, E-mail: amitkandhare25@gmail.com

http://dx.doi.org/10.17179/excli2018-1036

This is an Open Access article distributed under the terms of the Creative Commons Attribution License (http://creativecommons.org/licenses/by/4.0/).

Effect of hesperidin and insulin treatment on body weight (in gm) of diabetic rats

Rat No.	Normal	NWC	DWC	I (10)	H (25)	H (50)	H (100)
1	188.2	205.0	159.5	196.9	166.0	192.0	188.9
2	214.8	178.0	162.0	206.2	171.0	171.0	206.1
3	202.0	180.0	168.0	202.4	171.0	196.0	200.0
4	199.0	185.0	162.0	200.0	179.5	201.0	200.0
5	187.6	184.0	164.0	210.1	177.0	196.0	189.1
6	183.4	181.0	175.5	185.4	175.5	200.0	189.9

NWC: Normal wound control group; DWC: Diabetic wound control group; I (10): Insulin (10 IU/kg, s.c.) treated group; H (25): Hesperidin (25 mg/kg, p.o.) treated group; H (50): Hesperidin (50 mg/kg, p.o.) treated group; H (100): Hesperidin (100 mg/kg, p.o.) treated group

EXCLI Journal 2018;17:399-419 – ISSN 1611-2156
Received: January 07, 2018, accepted: March 28, 2018, published: May 04, 2018

Effect of hesperidin and insulin treatment on serum glucose level (in mg/dL) of diabetic rats

Rat No.	Normal	NWC	DWC	I (10)	H (25)	H (50)	H (100)
**1**	251.15	290.09	333.38	221.75	340.09	253.88	284.95
**2**	295.91	247.08	348.97	307.01	302.23	257.24	288.43
**3**	299.88	315.96	328.38	301.06	324.2	272.48	292.17
**4**	308.89	251.95	286.13	249.81	297.94	321.61	282.22
**5**	294.89	318.72	371.81	305.3	351.19	332.4	299.65
**6**	261.42	306.47	319.62	325.15	300.05	330.12	287.68

NWC: Normal wound control group; DWC: Diabetic wound control group; I (10): Insulin (10 IU/kg, s.c.) treated group; H (25): Hesperidin (25 mg/kg, p.o.) treated group; H (50): Hesperidin (50 mg/kg, p.o.) treated group; H (100): Hesperidin (100 mg/kg, p.o.) treated group

Effect of hesperidin and insulin treatment on food intake (in gm) of diabetic rats

Rat No.	Normal	NWC	DWC	I (10)	H (25)	H (50)	H (100)
**1**	49.7	59.6	72.4	33.3	64.5	59.3	59.9
**2**	40.5	32.9	60.0	36.0	64.9	29.0	52.9
**3**	49.7	40.0	76.4	46.0	77.9	59.7	36.2
**4**	42.1	46.6	74.2	21.9	63.6	56.1	48.6
**5**	43.5	48.7	72.5	36.6	54.3	55.3	57.8
**6**	53.8	60.9	77.3	37.5	61.8	53.3	48.3

NWC: Normal wound control group; DWC: Diabetic wound control group; I (10): Insulin (10 IU/kg, s.c.) treated group; H (25): Hesperidin (25 mg/kg, p.o.) treated group; H (50): Hesperidin (50 mg/kg, p.o.) treated group; H (100): Hesperidin (100 mg/kg, p.o.) treated group

Effect of hesperidin and insulin treatment on water intake (in ml) of diabetic rats

Rat No.	Normal	NWC	DWC	I (10)	H (25)	H (50)	H (100)
**1**	61.6	69.6	130.9	41.9	112.1	89.9	88.3
**2**	81.0	51.1	139.5	41.7	111.5	93.9	89.9
**3**	54.4	67.2	129.7	59.0	115.4	95.7	81.6
**4**	79.6	65.8	98.7	42.0	111.0	104.4	82.5
**5**	65.8	82.2	91.9	63.0	129.1	93.9	83.6
**6**	69.3	51.8	132	41.1	96.7	92.9	106.3

NWC: Normal wound control group; DWC: Diabetic wound control group; I (10): Insulin (10 IU/kg, s.c.) treated group; H (25): Hesperidin (25 mg/kg, p.o.) treated group; H (50): Hesperidin (50 mg/kg, p.o.) treated group; H (100): Hesperidin (100 mg/kg, p.o.) treated group

EXCLI Journal 2018;17:399-419 – ISSN 1611-2156
Received: January 07, 2018, accepted: March 28, 2018, published: May 04, 2018

Effect of hesperidin and insulin treatment on serum insulin (in mg/L) of diabetic rats

Rat No.	Normal	NWC	DWC	I (10)	H (25)	H (50)	H (100)
**1**	2.81	2.84	0.08	0.69	0.79	0.34	0.16
**2**	2.98	2.07	0.08	0.44	1.11	0.31	4.82
**3**	2.14	2.01	0.92	4.72	1.83	2.59	0.09
**4**	2.82	4.23	1.04	0.88	0.62	0.29	3.78
**5**	2.73	2.48	0.01	4.2	0.52	2.92	4.92
**6**	2.6	2.64	1.01	4.59	0.45	2.93	0.25

NWC: Normal wound control group; DWC: Diabetic wound control group; I (10): Insulin (10 IU/kg, s.c.) treated group; H (25): Hesperidin (25 mg/kg, p.o.) treated group; H (50): Hesperidin (50 mg/kg, p.o.) treated group; H (100): Hesperidin (100 mg/kg, p.o.) treated group

Effect of hesperidin and insulin treatment on % Wound Closure at day 21 of diabetic rats

Rat No.	Normal	NWC	DWC	I (10)	H (25)	H (50)	H (100)
**1**	---	6.1	-49.1	16.2	18.9	10.1	75.4
**2**	---	60.5	-1.8	92.4	3.4	46.3	147.7
**3**	---	5.2	-10.5	63.3	5.6	48.2	60.2
**4**	---	12.2	-15.9	54.9	20.2	78.9	39.1
**5**	---	37.2	-9.1	24.8	31.5	77.1	113.2
**6**	---	55.5	-47.7	50.4	13.7	11.1	145

NWC: Normal wound control group; DWC: Diabetic wound control group; I (10): Insulin (10 IU/kg, s.c.) treated group; H (25): Hesperidin (25 mg/kg, p.o.) treated group; H (50): Hesperidin (50 mg/kg, p.o.) treated group; H (100): Hesperidin (100 mg/kg, p.o.) treated group

Received: January 07, 2018, accepted: March 28, 2018, published: May 04, 2018

Effect of hesperidin and insulin treatment on SOD (in U/mg of protein) level of diabetic rats

Rat No.	Normal	NWC	DWC	Insulin	H (25)	H (50)	H (100)
1	9.407209	2.11146	3.160202	7.483663	2.621317	4.527345	7.565385
2	7.814116	4.238486	2.25775	8.025936	4.292101	6.841292	6.787508
3	7.848567	4.404932	2.12965	7.734881	3.459655	6.281398	5.073538
4	9.606504	5.724699	1.312348	6.302266	4.57976	5.35216	6.113248

NWC: Normal wound control group; DWC: Diabetic wound control group; I (10): Insulin (10 IU/kg, s.c.) treated group; H (25): Hesperidin (25 mg/kg, p.o.) treated group; H (50): Hesperidin (50 mg/kg, p.o.) treated group; H (100): Hesperidin (100 mg/kg, p.o.) treated group

Effect of hesperidin and insulin treatment on GSH (in µg/mg of protein) level of diabetic rats

Rat No.	Normal	NWC	DWC	Insulin	H (25)	H (50)	H (100)
1	4.141177	2.110403	2.10698	4.849003	2.546769	2.857635	4.385448
2	7.249683	2.895888	2.205837	5.112994	2.421569	3.853924	4.222222
3	6.418048	2.739018	2.26943	4.723204	2.044657	4.108322	5.940476
4	7.281046	2.34747	1.390868	7.74398	1.806548	4.489068	5.397553

NWC: Normal wound control group; DWC: Diabetic wound control group; I (10): Insulin (10 IU/kg, s.c.) treated group; H (25): Hesperidin (25 mg/kg, p.o.) treated group; H (50): Hesperidin (50 mg/kg, p.o.) treated group; H (100): Hesperidin (100 mg/kg, p.o.) treated group

Effect of hesperidin and insulin treatment on MDA (in nM/mg of protein) level of diabetic rats

Rat No.	Normal	NWC	DWC	Insulin	H (25)	H (50)	H (100)
1	4.558824	16.84713	22.6272	8.782051	20.55166	12.61634	8.775811
2	5.608365	15.96129	21.45161	7.944915	21.77696	14.62704	10.21318
3	7.838398	14.78682	19.62435	9.65106	17.61962	15.6539	13.63839
4	6.568627	13.44866	20.54632	8.65019	18.56027	19.46118	12.55734

NWC: Normal wound control group; DWC: Diabetic wound control group; I (10): Insulin (10 IU/kg, s.c.) treated group; H (25): Hesperidin (25 mg/kg, p.o.) treated group; H (50): Hesperidin (50 mg/kg, p.o.) treated group; H (100): Hesperidin (100 mg/kg, p.o.) treated group

Effect of hesperidin and insulin treatment on NO (in µg/mL) level of diabetic rats

Rat No.	Normal	NWC	DWC	Insulin	H (25)	H (50)	H (100)
1	10.75	31.3	62.6	40.8	48.75	41	39.85
2	12.45	27.95	58.65	55.95	40.1	31.35	36.25
3	21.2	45.8	50.45	50.55	55.45	50.6	31
4	14.65	32.55	60.5	45.6	62.5	46.5	26.25

NWC: Normal wound control group; DWC: Diabetic wound control group; I (10): Insulin (10 IU/kg, s.c.) treated group; H (25): Hesperidin (25 mg/kg, p.o.) treated group; H (50): Hesperidin (50 mg/kg, p.o.) treated group; H (100): Hesperidin (100 mg/kg, p.o.) treated group

Effect of hesperidin and insulin treatment on the hydroxyproline (in µg/mg tissue) level of diabetic rats

Rat No.	Normal	NWC	DWC	Insulin	H (25)	H (50)	H (100)
**1**	4.209091	2.018182	0.8909091	1.890909	0.7818182	3.018182	4.518182
**2**	4.327273	1.209091	0.7545455	2.881818	0.9545454	2.772727	3.018182
**3**	3.518182	1.554545	0.8545455	1.018182	1.018182	2.618182	2.509091
**4**	5.618182	1.2	1.454545	2.554545	1.436364	1.518182	2.436364

NWC: Normal wound control group; DWC: Diabetic wound control group; I (10): Insulin (10 IU/kg, s.c.) treated group; H (25): Hesperidin (25 mg/kg, p.o.) treated group; H (50): Hesperidin (50 mg/kg, p.o.) treated group; H (100): Hesperidin (100 mg/kg, p.o.) treated group

Effect of hesperidin and insulin treatment on mRNA expression of VEGF-c in wound tissue

Rat No.	Normal	NWC	DWC	Insulin	H (25)	H (50)	H (100)
**1**	2.54	2.03	0.52	2.55	1.65	2.66	2.66
**2**	2.66	1.58	0.89	2.63	0.96	1.54	3.01
**3**	2.89	1.11	0.76	2.78	0.88	1.85	2.78
**4**	3.15	1.05	0.39	1.96	0.85	1.95	1.62

NWC: Normal wound control group; DWC: Diabetic wound control group; I (10): Insulin (10 IU/kg, s.c.) treated group; H (25): Hesperidin (25 mg/kg, p.o.) treated group; H (50): Hesperidin (50 mg/kg, p.o.) treated group; H (100): Hesperidin (100 mg/kg, p.o.) treated group

Effect of hesperidin and insulin treatment on mRNA expression of Ang-1 in wound tissue

Rat No.	Normal	NWC	DWC	Insulin	H (25)	H (50)	H (100)
**1**	3.21	1.52	0.56	2.78	1.56	1.56	2.33
**2**	2.52	1.86	0.42	3.02	1.12	0.89	2.63
**3**	3.32	2.04	0.63	2.88	0.48	1.48	1.98
**4**	3.52	2.33	0.78	2.56	0.78	2.63	2.63

NWC: Normal wound control group; DWC: Diabetic wound control group; I (10): Insulin (10 IU/kg, s.c.) treated group; H (25): Hesperidin (25 mg/kg, p.o.) treated group; H (50): Hesperidin (50 mg/kg, p.o.) treated group; H (100): Hesperidin (100 mg/kg, p.o.) treated group

Effect of hesperidin and insulin treatment on mRNA expression of Tie-2 in wound tissue

Normal	NWC	DWC	Insulin	H (25)	H (50)	H (100)
2.56	0.89	0.56	1.55	0.56	1.87	2.66
2.48	1.22	0.32	2.03	0.23	0.63	2.52
2.96	1.63	0.11	1.48	0.48	1.46	2.12
3.11	1.25	0.45	1.52	0.96	1.23	2.65

NWC: Normal wound control group; DWC: Diabetic wound control group; I (10): Insulin (10 IU/kg, s.c.) treated group; H (25): Hesperidin (25 mg/kg, p.o.) treated group; H (50): Hesperidin (50 mg/kg, p.o.) treated group; H (100): Hesperidin (100 mg/kg, p.o.) treated group

EXCLI Journal 2018;17:399-419 – ISSN 1611-2156
Received: January 07, 2018, accepted: March 28, 2018, published: May 04, 2018

Effect of hesperidin and insulin treatment on mRNA expression of TGF-β in wound tissue

Rat No.	Normal	NWC	DWC	Insulin	H (25)	H (50)	H (100)
**1**	2.15	1.63	0.23	1.11	0.23	1.22	1.63
**2**	2.63	0.98	0.35	2.74	0.85	0.62	1.52
**3**	2.78	1.52	0.42	2.08	0.63	0.87	2.01
**4**	2.02	1.45	0.15	1.63	0.45	1.52	2.56

NWC: Normal wound control group; DWC: Diabetic wound control group; I (10): Insulin (10 IU/kg, s.c.) treated group; H (25): Hesperidin (25 mg/kg, p.o.) treated group; H (50): Hesperidin (50 mg/kg, p.o.) treated group; H (100): Hesperidin (100 mg/kg, p.o.) treated group

Effect of hesperidin and insulin treatment on mRNA expression of Smad 2/3 in wound tissue

Rat No.	Normal	NWC	DWC	Insulin	H (25)	H (50)	H (100)
**1**	1.65	0.62	0.25	1.22	0.56	1.25	1.64
**2**	2.63	0.63	0.32	1.55	0.95	1.48	1.62
**3**	1.52	0.45	0.26	0.85	0.75	1.23	2.02
**4**	1.77	0.88	0.59	0.63	0.66	1.22	1.62

NWC: Normal wound control group; DWC: Diabetic wound control group; I (10): Insulin (10 IU/kg, s.c.) treated group; H (25): Hesperidin (25 mg/kg, p.o.) treated group; H (50): Hesperidin (50 mg/kg, p.o.) treated group; H (100): Hesperidin (100 mg/kg, p.o.) treated group
